# Simulated Microgravity Modulates Focal Adhesion Gene Expression in Human Neural Stem Progenitor Cells

**DOI:** 10.3390/life12111827

**Published:** 2022-11-09

**Authors:** Wei Wang, Elena Di Nisio, Valerio Licursi, Emanuele Cacci, Giuseppe Lupo, Zaal Kokaia, Sergio Galanti, Paolo Degan, Sara D’Angelo, Patrizio Castagnola, Sara Tavella, Rodolfo Negri

**Affiliations:** 1 Department of Biology and Biotechnologies “C. Darwin”, Sapienza University of Rome, 00185 Rome, Italy; 2 Institute of Molecular Biology and Pathology (IBPM), National Research Council (CNR) of Italy c/o Department of Biology and Biotechnologies “C. Darwin”, Sapienza University, 00185 Rome, Italy; 3 Lund Stem Cell Center, Department of Clinical Sciences, Lund University, 22184 Lund, Sweden; 4 Excise, Custom and Monopolies Agency, ADM, 00153 Rome, Italy; 5 IRCCS Ospedale Policlinico San Martino, 16132 Genova, Italy; 6 Department of Experimental Medicine (DIMES), University of Genoa, 16132 Genoa, Italy

**Keywords:** microgravity, space conditions, human neural stem progenitor cells, focal adhesion, laminin

## Abstract

We analyzed the morphology and the transcriptomic changes of human neural stem progenitor cells (hNSPCs) grown on laminin in adherent culture conditions and subjected to simulated microgravity for different times in a random positioning machine apparatus. Low-cell-density cultures exposed to simulated microgravity for 24 h showed cell aggregate formation and significant modulation of several genes involved in focal adhesion, cytoskeleton regulation, and cell cycle control. These effects were much more limited in hNSPCs cultured at high density in the same conditions. We also found that some of the genes modulated upon exposure to simulated microgravity showed similar changes in hNSPCs grown without laminin in non-adherent culture conditions under normal gravity. These results suggest that reduced gravity counteracts the interactions of cells with the extracellular matrix, inducing morphological and transcriptional changes that can be observed in low-density cultures.

## 1. Introduction

More than fifty years of manned spaceflight have shown that space is a hostile environment for human health. Factors, such as gravity alterations, ionizing radiation, perturbation of circadian rhythm, confinement, and isolation, are the main characterized challenges for astronauts’ health [1,2]. Understanding the consequences of these sources of stress on human physiology is a major challenge to ensure successful and safe space missions [2,3]. Previous research has mainly focused on the cardiovascular and musculoskeletal systems, showing that space travel is associated with the alteration of these systems, such as muscle atrophy [4] and bone density loss [5]. Less attention has been given to the effects on the human nervous system, although several neurological alterations have been observed in astronauts returning from space missions, such as psychological and behavioral problems [6], cephalic fluid shifts, visual impairment due to spaceflight-associated neuro-ocular syndrome (SANS), neurovestibular problems [7], cognitive alterations [1,3,7], gray matter changes, and white matter decline [8]. During space flights, astronauts are subjected to multiple inputs, including psychological stress, high workload, lack of sleep, and physiological changes, but reduced gravity is likely the main condition involved in nervous system alterations. Nevertheless, the mechanisms and the neural substrates involved in these effects remain unclear. Studies with animals showed that long permanence in space environments caused alterations in cerebral cortex development and architecture in rats [9], along with wide transcriptomic changes in the mouse hippocampus and hypothalamus [6,10]. These studies, although suggestive, are seriously hampered by the low number of animals analyzed and by the long time required to recover and analyze them. It is also difficult to separate the effects of the different stress sources associated with space travel. Experiments performed by keeping animals in conditions of simulated hypergravity [11,12] or microgravity [13] confirmed the induction of transcriptional reprogramming in different brain regions, but, in these same studies, the action of additional stress sources and concomitant physiological alterations in other body organs and districts could not be ruled out. Cellular models are, therefore, required to better understand the effects of microgravity on neural cells. Several studies have been performed on mammalian stem cells to analyze the influence of simulated microgravity on their proliferation and differentiation capability.

Chen et al. [14] showed that three days of cultivation in a rotating vessel (RCCS) in simulated microgravity conditions enhanced the differentiation of rat mesenchymal stem cells into neurons. To assess whether exposure to reduced gravity could be beneficial for hNSPCs in vitro expansion, Chiang et al. [15] maintained these cells for 72 h in an RCCS apparatus (0.01 g). This study showed that simulated microgravity induces alterations in mitochondrial activity and cell proliferation associated with the activation of intracellular signaling and metabolic pathways [15].

In this work, we present a morphological and transcriptomic analysis of hNSPCs, grown as adherent monolayers on a laminin matrix and kept for up to 24 h in conditions of simulated microgravity in a random positioning machine (RPM) apparatus. Our results suggest that reduced gravity counteracts the interactions of cells with the extracellular matrix, thus, inducing morphological changes and feedback regulation of genes involved in focal adhesion and cell cycle. These phenotypes were not evident when the cells were grown to confluence, likely due to stronger cell–matrix interactions.

## 2. Materials and Methods

### 2.1. Cell Cultures and Exposure to Simulated Microgravity Conditions

The human neural progenitor cell line used in these experiments was derived from the spinal cord of a 9-week post-conception fetus. Fetuses were obtained from Lund and Malmö University Hospitals, according to guidelines approved by the Lund/Malmö Ethical Committee. Informed consent for the donation of fetal tissue was obtained from the mother.

The hNSPCs were expanded under culture conditions previously described (Ajmone Cat et al., 2019). Briefly, cells were cultured in DMEM/F12 medium supplemented with 50× B27 minus vitamin A (Gibco, by Thermo Fisher Scientifc, Waltham, MA, USA), 100× P/S, 100× Glutamax, 10 ng/mL bFGF (R&D Systems, Minneapolis, MN, USA), 10 ng/mL EGF (R&D Systems, Minneapolis, MN, USA), and 1 mM Hepes.

The hNSPCs grown under adherent conditions were plated on 0.1 μg/mL poly-L-ornithine and 10 μg/mL laminin-coated flasks T75 Cell Culture Flask, Canted Neck Plug Seal Cap (Corning, Life Sciences, NY, USA, 430720), while hNSPCs forming neurospheres were plated on uncoated flasks. Densities of 10,000 cells/cm^2^ for the low-density condition and 20,000–40,000 cells/cm^2^ for the high-density condition were seeded five days before the experiments in simulated microgravity. Cell culture in simulated microgravity was accomplished in a random positioning machine (RPM; Dutch Space, Leiden, NL, The Netherlands) located in a dedicated 37 °C controlled room. The instrument consists of two independently rotating frames. The spinning velocity of the frames was set at 60 degrees s^−1^. The separate rotation of each frame was random and driven by dedicated software to obtain a constant simulation at the chosen value of the g vector, accordingly to the manufacturer’s instructions. For the routine conditions employed in our experiments, we set a value of 0.003 g.

In the microgravity simulation experimental paradigm, hNSPCs were plated in coated flasks filled with the culture medium to avoid air bubbles and foam formation during RPM exposure for 6 or 24 h. Control cultures were maintained in the same culture condition, except for RPM exposure. After 6 h or 24 h, the cells were detached using accutase (Corning Life Sciences, Corning, NY, USA) and counted with Trypan blue using the TC20 Automated Cell Counter (Bio-Rad, Hercules, CA, USA). In particular, the sham cells were placed in the same chamber on a mechanical agitator Model 3005 from GFL (Burgwedel, Germany) with non-orbital motion set at a rotation speed of 100 rpm to exclude any possible contribution of the 2D motion component. At the end of the experiment cell, culture images were taken under a Leica DMi1 microscope.

### 2.2. RNA-Seq Analysis

For RNA sequencing (RNA-Seq), total RNA was purified from hNSPC cultures using QIAGEN RNeasy kits (QIAGEN, 74004, Venlo, The Netherlands). RNA-Seq libraries from the total RNA (100 ng) from each sample were prepared using a QuantSeq 3′ mRNA-Seq Library prep kit (Lexogen, Vienna, Austria), according to the manufacturer’s instructions, at Next Generation Diagnostics (Pozzuoli, Italy). The amplified fragmented cDNAs of 300 bp in size were sequenced in single-end mode using the NextSeq500 (Illumina, San Diego, CA, USA) with a read length of 101 bp. The sequence read quality was evaluated using FastQC v.0.11.8 (Babraham Institute, Cambridge, UK), followed by trimming using BBDuk software to remove adapter sequences, poly-A tails, and low-quality end bases (Q < 20). Reads were then mapped to the human genome reference build GRCh38 (obtained from Ensembl database) using STAR v.2.5.0a [16]; gene annotations corresponding to Ensembl annotation release 96 were used to build a transcriptome index, which was provided to STAR during the alignment.

### 2.3. Differential Gene Expression Analysis and Functional Enrichment Analysis

To identify differentially expressed genes (DEGs), data were filtered to remove the genes with a <1 count per million in less than 7 out of 16 total samples for each comparison from the analysis. DEGs were assessed by comparing control and hNSPC samples exposed to simulated microgravity using a moderated t-test with a false discovery rate (FDR) threshold of <0.1. Data normalization and differential gene expression analysis were performed using Bioconductor R package DESeq2 v.1.34 [17,18].

DEGs were clustered by functional annotation in gene ontology and pathway enrichment analysis using Bioconductor R package clusterProfiler v.4.2.0 [19] with annotation of Gene Ontology Database and with annotation of Kyoto Encyclopedia of Genes and Genomes (KEGG) [20] for pathways. The R package pathview v.1.34.0 was used to integrate the RNA-Seq data with KEGG pathway plots.

### 2.4. Real-Time Quantitative PCR

Total mRNA was isolated using the miRNeasy^®^ Mini Kit (QIAGEN, 166034218, Venlo, The Netherlands) and then transcribed to cDNA using SensiFAST cDNA Synthesis Kit (Meridian Bioscience, BIO-65054). RT-qPCR was performed in a 20 μL reaction system containing forward and reverse primers, cDNA, and SensiFAST SYBR Hi-ROX (Meridian Bioscience, BIO-92020, UK) using a 96-well plate (FrameStar^®^ FastPlate 96, 4ti-0910/C) and StepOnePlus instrument (Thermo Fisher Scientific, Waltham, MA, USA). Two-step amplification (95 °C for 5 s and 60 °C for 30 s) was performed for 40 cycles. The target mRNA levels were calculated via the 2^−ΔΔCt^ method, using RPL27 as a reference gene. The primer sequences are listed in Table S1.

## 3. Results

### 3.1. Exposure to Simulated Microgravity Induces Morphological Changes in hNSPCs Grown at a Low Cell Density

**Figure 1:**
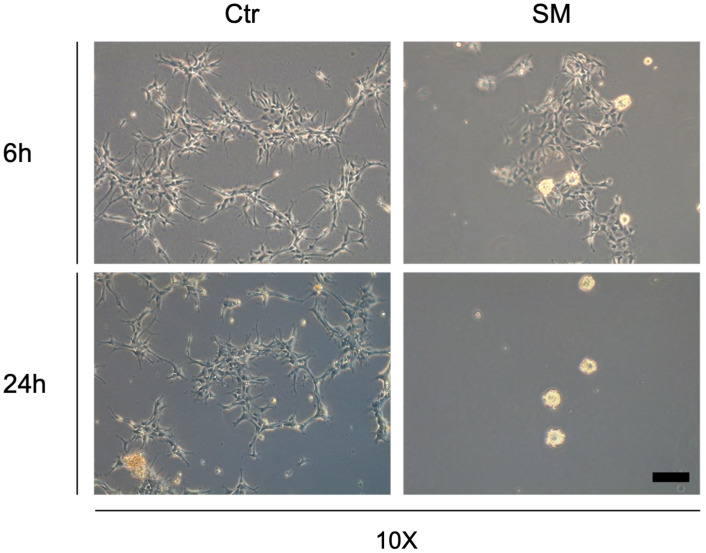
Phase contrast microscopy images of hNSPCs in normal conditions or exposed to 6 or 24 h of simulated microgravity (SM). Scale bar 100 µm.

The hNSPCs were plated in T75 mL flasks coated with a matrix consisting of poly-L-ornithine and laminin, at a density of 10,000 cells/cm^2^, and cultured for 5 days at 37 °C in proliferation-supporting media, as described in Section 2. The flasks were then transferred to the RPM in a temperature-controlled room (37 °C) and kept in simulated microgravity for 6 h (6 h) or 24 h. Sham controls were kept in the same room at 1 g for the same time intervals. Two flasks were used for each treatment condition and time point in each experiment. We first morphologically analyzed the cultures by phase-contrast microscopy. Although no signs of cell detachment or aggregate formation were observed in the sham cultures, nor in all cultures at time 0, incubation in simulated microgravity conditions induced dramatic morphological changes, as previously observed for other human cell types [21,22,23,24,25], leading to the formation of “floating cell clumps”. These clumps were barely visible at 6 h but involved a significant fraction of the cell population at 24 h (Figure 1). These morphological effects were not associated with evident changes in cell viability or cell proliferation. In particular, the live cell fraction, as assessed by trypan blue exclusion assays, was comparable between the RPM-treated and control cultures at 24 h (Table 1). Moreover, no significant alterations in the fractions of cells in G1, S, and G2/M cycle phases were observed among the different experimental conditions following flow cytometry of DAPI-stained cells (Figure S1).

### 3.2. Exposure to Simulated Microgravity induces Changes in the hNSPCs Transcriptome

**Figure 2:**
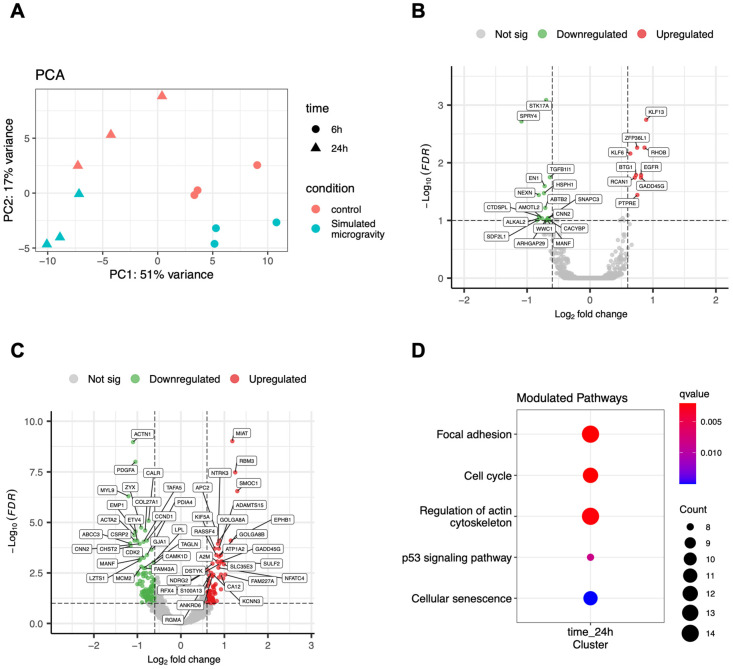
(**A**) Principal component analysis of transcriptomic data of controls and samples exposed to simulated microgravity for 6 or 24 h (Table 1, experiments 1–3). (**B**) Volcano plots showing genes differentially expressed in samples exposed to simulated microgravity for 6 h. (**C**) Volcano plots showing genes differentially expressed in samples exposed to simulated microgravity for 24 h; (**D**) KEGG pathways significantly enriched after 24 h of simulated microgravity.

**Figure 3:**
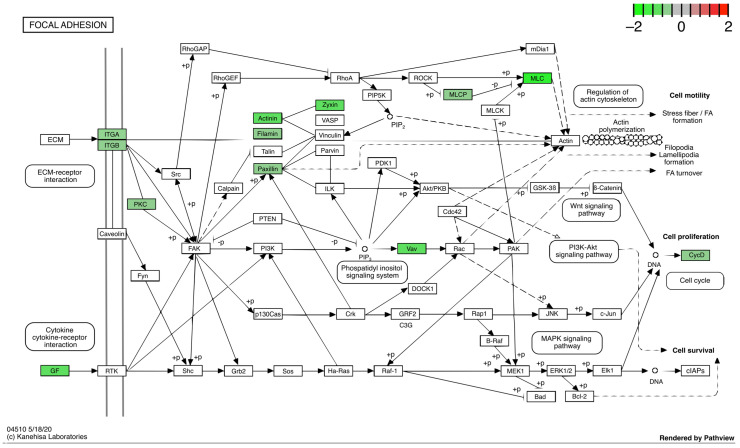
DEGs at 24 h comparison that belong to focal adhesion KEGG pathway. Down-regulated genes are colored in green; no genes of this pathway were significantly up-regulated.

To analyze the transcriptomic changes induced by simulated microgravity, we extracted the total RNA from all the experimental samples and performed RNA-Seq analysis (see Materials and Methods). Figure 2A shows a PCA analysis of the global transcriptome variability between the control and simulated microgravity conditions; it is evident that the samples are clustered in the first component based on the time (6 h vs. 24 h) and in the second component based on the treatment (SM vs. sham). We then proceeded with statistical analysis of the data to determine the genes significantly modulated by simulated microgravity (FDR < 0.1). Using a false discovery rate of 0.1 (FDR < 0.1) and a fold change of 1.5 ($\left|{\mathrm{Log}}_{2}\mathrm{FC}\right|$ > 0.58) as thresholds, this analysis identified 28 differentially expressed genes (DEGs) between control and RPM-treated cultures at 6 h and 207 DEGs at 24 h (Figure 2B,C, Figures S2 and S3 and Table S2). The ontological analysis of the DEG list identified the following enriched biological processes: focal adhesion, regulation of the actin cytoskeleton, and cell cycle (Figure 2D). Strikingly, we found that, among the metabolic pathways, focal adhesion was the most enriched, with several genes of that pathway negatively modulated by simulated microgravity (Figure 3). The pathway of cytoskeleton regulation was also significantly enriched in modulated genes (Figure S3).

### 3.3. HNSPCs Growth in Non-Adherent Conditions Partially Recapitulates the Transcriptomic Effects of Simulated Microgravity

**Figure 4:**
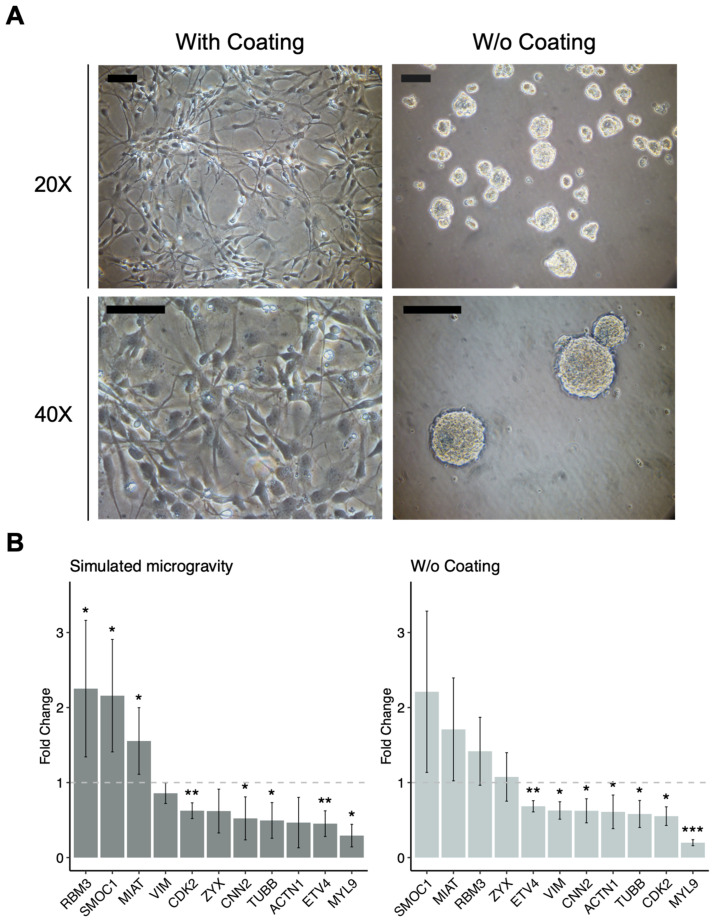
(**A**) Human NSPCs in adherent and normal gravity conditions (on the left); human NSPCs in non-adherent and normal gravity conditions (on the right). Scale bars 100 µm; (**B**) Relative expression of selected target genes in simulated microgravity and no-coating conditions. The standard deviation is indicated. *p*-values of statistical significance were calculated by Student’s *T*-test: * <0.05; ** <0.01; *** <0.001.

A group of DEGs involved in focal adhesion and cell cycle regulation was selected for quantitative RT-PCR (RT-qPCR) validation of the transcriptomic results. Figure 4 and Figure S4A show an excellent correlation between RNA-Seq and RT-qPCR data for the selected DEGs. Next, we evaluated the relevance of hNSPC adhesion to a laminin-coated substrate in regard to the observed transcriptional modulations. For this purpose, we tested the same selected DEGs in hNSPCs, plated as single cells on uncoated flasks, and grown in proliferation-supporting media with normal gravity for 5 days, until they formed floating cell aggregates. Gene expression levels were compared with those from adherent cultures of hNSPCs plated at the same density but grown in flasks coated with poly-L-ornithine and laminin (Figure 4A). Notably, hNSPC culture in non-adherent and normal gravity conditions induced similar gene expression changes to those detected upon RPM treatment, in comparison with adherent and normal gravity conditions (Figure 4B and Figure S4B).

### 3.4. Growth of hNSPCs at High Cell Density in Simulated Microgravity Conditions Causes Limited Morphological and Transcriptomic Changes

**Figure 5:**
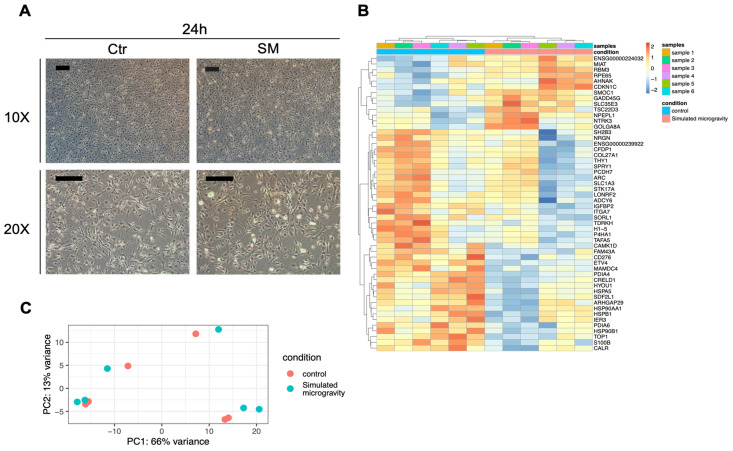
(**A**) Phase contrast microscopy images of human NSPCs grown at higher density in normal conditions (Ctr) or exposed to 6 or 24 h of SM. In this case, no significant morphological changes were observed between the two conditions. The scale bars are equal to 100 µm. (**B**) Heatmap showing the scaled expression levels (z-score) of the target genes that changed significantly in hNSPCs grown at higher density in simulated microgravity conditions. (**C**) Principal component analysis (PCA) of the transcriptomic profiles of all experiments performed at 24 h (Table 1, experiments 1–6).

When we exposed higher density adherent hNSPC cultures to simulated microgravity, we did not observe the same results. For these experiments, hNSPCs were plated at a density of 20,000–40,000 cells/cm^2^ (see Table 1) in T75 mL flasks coated with poly-L-ornithine and laminin and cultured for 5 days at 37 °C, followed by RPM or sham treatment for 6 h or 24 h, as described above. No significant morphological changes were observed (Figure 5A). Moreover, the transcriptome of high-density hNSPC cultures appeared very different compared to that of the less dense cultures, independent of exposure to simulated microgravity (Figure 5C). Indeed, in a complete analysis encompassing all 12 analyzed samples at 24 h (low-density and high-density cultures in simulated microgravity and normal gravity conditions) only 53 DEGs were found between control and RPM-treated samples with FDR < 0.1 (Figure 5B). Although some of the previously validated genes (SMOC1, RBM3, MIAT, and ETV4) were modulated by RPM treatment regardless of cell density, some of the focal adhesion genes were not modulated (MYL9, ACTN, TUBB, VIM) by simulated microgravity in the high-density cultures (Figure S4C).

## 4. Discussion

**Figure 6:**
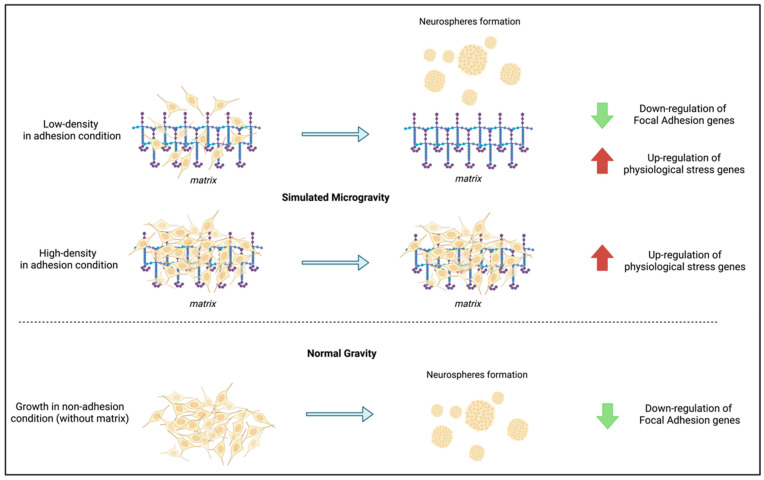
Diagram summarizing the main effects of simulated microgravity and/or absence of laminin matrix on human NSPCs.

It has been previously shown that normal and cancer human cell types cultured in simulated microgravity for several hours using an RPM apparatus undergo dramatic morphological changes, leading to two alternative phenotypes, adherent cells, and floating cell clumps, simultaneously present in the same culture [21,22,23,24,25,26]. The formation of floating clumps was also observed upon microgravity simulation in rotating vessels [27]. This is a reversible process; when a floating aggregate cell population is seeded in normal gravity, it goes back to a fully adherent phenotype; when reseeded in microgravity condition, it gives rise to the two above-mentioned phenotypes [25]. We observed similar behavior when growing hNSPCs in flasks coated with poly-L-ornithine and laminin at low density. The interaction of neural progenitor cells with the extracellular matrix was previously shown to be crucial for their initial morphological development and process outgrowth [28]. We showed that, when subjected to microgravity simulation in an RPM apparatus, hNSPCs progressively switch from a laminin-adherent phenotype to the formation of floating cell aggregates, involving at least 60% of the whole population after 24 h. This morphological change was accompanied by transcriptomic changes (mainly down-regulation), involving several genes belonging to the focal adhesion pathway. On the one hand, focal adhesion components were previously considered as biomechanical sensors of the microenvironment [29], and some focal adhesion genes were found to be modulated in human bone marrow mesenchymal stromal cells after different periods of simulated microgravity [30]. On the other hand, a significant general down-regulation of the pathway following perturbation of cell–matrix interactions was never described. This observation suggested the hypothesis that the transcriptomic changes could be due to a feedback transcriptional regulation of the focal adhesion pathway in response to the weakening of cell interactions with the extracellular matrix, which, in turn, was causing cell detachment and floating aggregate formation. We, therefore, tested the expression of a group of the genes modulated by RPM treatment in hNSPC cultures grown without the extracellular matrix, but with normal gravity, and we found a very similar modulation of most of the genes, including down-regulated focal adhesion genes (TUBB, ACTN, MYL9, VIM, ZYX). It would be important to understand if these gene modulations can be observed on most of the cells subjected to simulated microgravity or only on those that form the aggregates. This would require a separate analysis of aggregates formed at early time points of cultures. Interestingly, experiments in which hNSPCs were grown closer to confluence and subsequently subjected to simulated microgravity conditions did not show significant floating aggregate formation and significant modulation of focal adhesion genes. Our interpretation is that in dense cultures, cell–cell interactions are stronger and can successfully counteract the weakening of cell–matrix interactions due to simulated microgravity (Figure 6). It should be taken into account that detailed studies on fluid dynamics during RPM experiments showed other effects that could be involved in the observed phenotypes [31,32]. Shear stress and increased cell deformations could be caused by the stationary motion of the fluid-filled culture flask during random rotation, which could affect cell–matrix interactions, especially in low density cultures. To exclude any possible contribution of the 2D motion component, the sham control cultures were placed on a mechanical agitator with non-orbital motion set at a rotation speed of 100 rpm. A transcriptomic comparison between samples kept at normal gravity in agitation for 24 h samples vs. 6 h samples showed that most of the genes, which are differentially expressed after 24 h in simulated microgravity vs. sham controls, are not significantly modulated. There is an overlap of only 28 genes significantly modulated at 24 h vs. 6 h in sham controls and those modulated at 24 h of simulated microgravity (Figure S5). Moreover, the fold changes observed are in the opposite direction in 25 genes out of 28. This supports a specific effect of simulated microgravity. In any case, we cannot completely rule out the role of these effects and further work, including on-flight experiments or the use of different platforms of simulated microgravity, that will be required to measure their relevance.

Our results represent the first evidence that the effects of simulated microgravity on the structure of the cell network can coherently regulate gene expression, warranting further investigation of the regulatory mechanisms mediating these effects. In this regard, we analyzed the transcription factor enrichment in the promoters of down-regulated genes at 24 h of simulated microgravity and found several plausible candidates (Figure S6). Among them, we found KFL6 (Kruppel-like factor 6), which, together with KFL13, was among the few genes up-regulated after 6 h of simulated microgravity. These transcription factors play key roles in nervous system development and function [33]. A global transcriptomic analysis of the six cultures kept for 24 h in simulated microgravity showed reproducible up-regulation of RBM3, MIAT, and GADD45G. RBM3 and the LncRNA MIAT are very powerful regulators that appear to be induced by cell stress conditions, such as hypoxia and hypothermia [34,35]. They are both well expressed in brain cells and linked to oncogenic and pathological states. MIAT overexpression is also considered as a potent cardiovascular disease-promoting element [36]. GADD45G is induced by genotoxic damage and involved in the inhibition of cell cycle and cell migration [30]. Further analysis will be required to better understand their regulatory role upon simulated microgravity exposure. Finally, recent studies showed a major reshaping of chromatin domains with wide transcriptomic effects following short gravity alterations [37,38], which could also be mediated by structural changes in the cytoskeleton [38,39].

## 5. Conclusions

We showed that simulated microgravity can modulate the interaction of hNSPCs with a laminin-based extracellular matrix, leading to cell detachment and transcriptional modulation of cell adhesion and stress-related genes. Since this interaction has a crucial role in regulating the processes of hNSPC proliferation and differentiation and consequently on the activity of neurogenic niches, this line of research can become relevant for a deeper understanding of the effects of reduced gravity on the human nervous system and on stem cell niches in general.

## Figures and Tables

**Table 1:** The table reports the data for cells plated at a density of 10,000 cells/cm^2^ (experiments 1–3) or at a density of 20,000 (experiments 4 and 5) or 40,000 cells/cm^2^ (experiment 6), NA = Not Analyzed.

Condition	Experiment	Time	Cell/cm^2^	Neurospheres	Vitality
Ctr	1	6 h	22,000	No	59%
Ctr	1	24 h	20,800	No	56%
SM	1	6 h	16,000	<10%	62%
SM	1	24 h	11,105	>60%	55%
Ctr	2	6 h	19,260	No	92%
Ctr	2	24 h	33,360	No	85%
SM	2	6 h	13,192	<10%	92%
SM	2	24 h	19,850	>60%	85%
Ctr	3	6 h	26,315	No	84%
Ctr	3	24 h	41,650	No	92%
SM	3	6 h	22,080	<10%	89%
SM	3	24 h	25,775	>60%	88%
Ctr	4	24 h	41,900	No	NA
SM	4	24 h	46,966	<5%	NA
Ctr	5	24 h	33,533	No	NA
SM	5	24 h	38,266	<5%	NA
Ctr	6	24 h	86,333	No	92%
SM	6	24 h	82,333	No	90%

## Data Availability

Raw count data of RNA-Seq are provided in Table S3.

## Supplementary Materials

The following supporting information can be downloaded at: https://www.mdpi.com/article/10.3390/life12111827/s1, Figure S1: cytofluorometric analysis; Figure S2: MA-plots of RNA-Seq; Figure S3: DEGs involved in Regulation of Actin cytoskeleton pathway; Figure S4: validation of RNA-Seq data; Figure S5: Comparison of DEGs between normal gravity samples (24 h vs. 6 h) and 24 h in simulated microgravity vs. sham controls comparisons; Figure S6: TF prediction results; Table S1: primers sequences; Table S2: significantly DEGs found by RNA-Seq; Table S3: raw counts data of RNA-Seq.
