# Supplementary material for: Simulated Microgravity Modulates Focal Adhesion Gene Expression in Human Neural Stem Progenitor Cells
Source: Life (Basel). 2022 Nov 9;12(11):1827. doi: 10.3390/life12111827 (PMC9699612; doi:10.3390/life12111827)
Supplement: Supplementary file 1 [file life-12-01827-s001.zip › Table S1.pdf]

Table S1. Primers Sequences

Target Genes	Forward Primer (5' to 3')	Reverse Primer (5' to 3')
MIAT	AGTTTGAGATGGGAGGCGCT	TGGTTACCCCTGTGATGCC
ACTN1	CATCAACAATGCCTGGGGCT	CCTCTTTGCCGTCAGTCCAG
PDGFA	TAGAGGAGCATTTGGAGTGCG	CATCTGGTTGGCTGCTTTAGG
RBM3	CCTATCTCTGAGGTGGTCGTT	CTCTCCGTTCATGGCTCTCA
SMOC1	ATGGCTCCTTTACCCAGGTG	ACCCGTCATCTTTCCTTCCTG
MYL9	CCCACCAGAAGCCAAGATGT	CTGTGGGGTTCTTCCCCAG
ZYX	GTGTCCCCGACTACCACAAG	CCTCAATCGACAGGGGCTTC
ETV4	AGTGCCTTTACTCCAGTGCCT	GGAGGTGCCAGAGGATCTCA
CNN2	TGGCACCAGCTAGAAAACCTG	CGAGAAGAGACACCTGCACC
CDK2	CTTTGCTGAGATGGTGACTCG	TCATCCAGGGGAGGTACAACT
TUBB	GGACCGCATCTCTGTGTACT	GCCCCAGACTGACCAAATAC
VIM	GGACCAGCTAACCAACGAC	GACGCATTGTCAACATCCTG
ITGA7	TCTGTGGAGACGCCATGTTC	CAGGAAGTGGATCTCTGCCC
KLF6	CCACTTGAAAGCACACCAGC	AGACCTGGAAAAACACCTGTCA
KLF13	CACACAGGTGAGAGGCCCTT	CATTCCCGGGTGGAAGTTGG
RPL27	ACCGGGGTGAAAGAAATGGG	GGTCAATTCCAGCCACCAGA
