# Supplementary material for: Simulated Microgravity Modulates Focal Adhesion Gene Expression in Human Neural Stem Progenitor Cells
Source: Life (Basel). 2022 Nov 9;12(11):1827. doi: 10.3390/life12111827 (PMC9699612; doi:10.3390/life12111827)
Supplement: Supplementary file 1 [file life-12-01827-s001.zip › Wang Di Nisio et al Supplementary figures - Revision3.pdf]

Figure S1

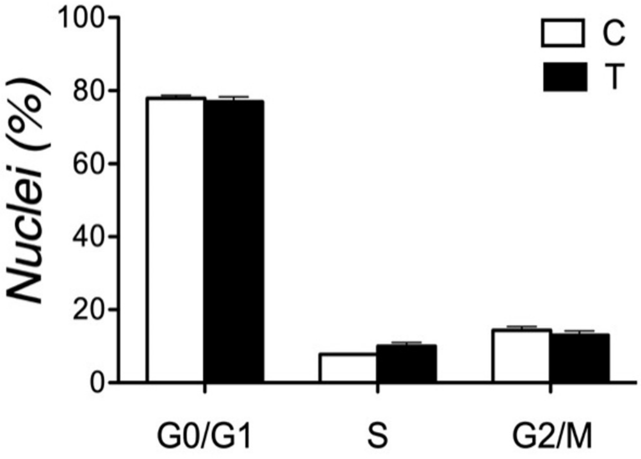

Figure S1. Histogram reporting the results of cytofluorimetric analysis of human NSPCs exposed to simulated microgravity (n=3).

Figure S2

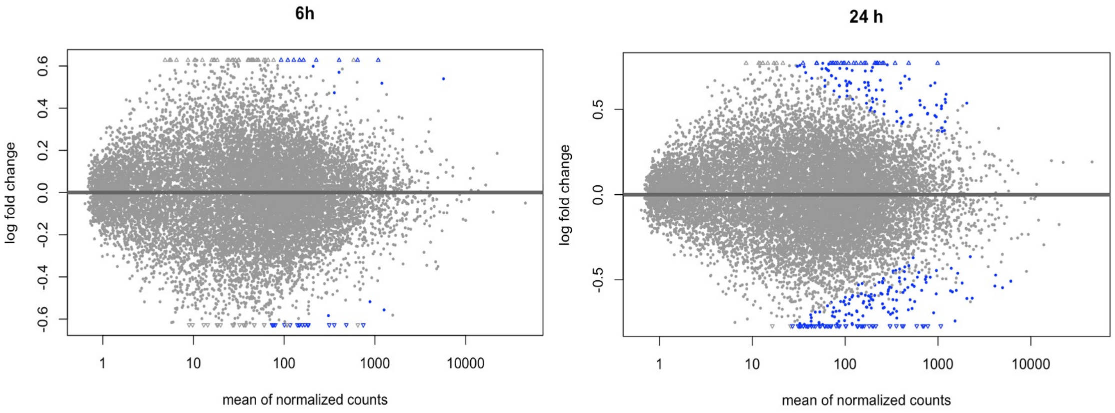

Figure S2. MA plot of the RNA-Seq data on hNSPCs exposed to 6 or 24 hours of simulated microgravity, respectively. Significantly DEGs (FDR < 0.1) are colored in blue.

Figure S3

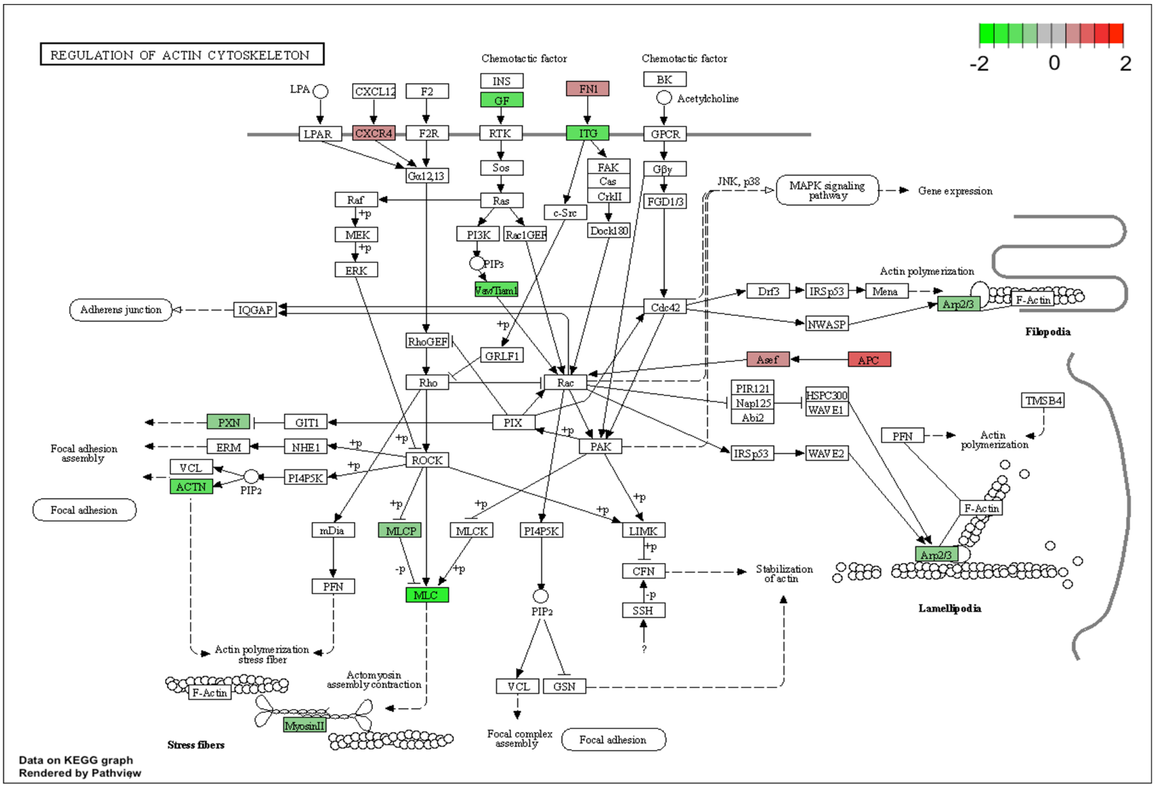

Figure S3, Genes significantly modulated in Regulation of Actin cytoskeleton KEGG pathway. Down-regulated genes are colored in green, up-regulated genes are in red.

Figure S4

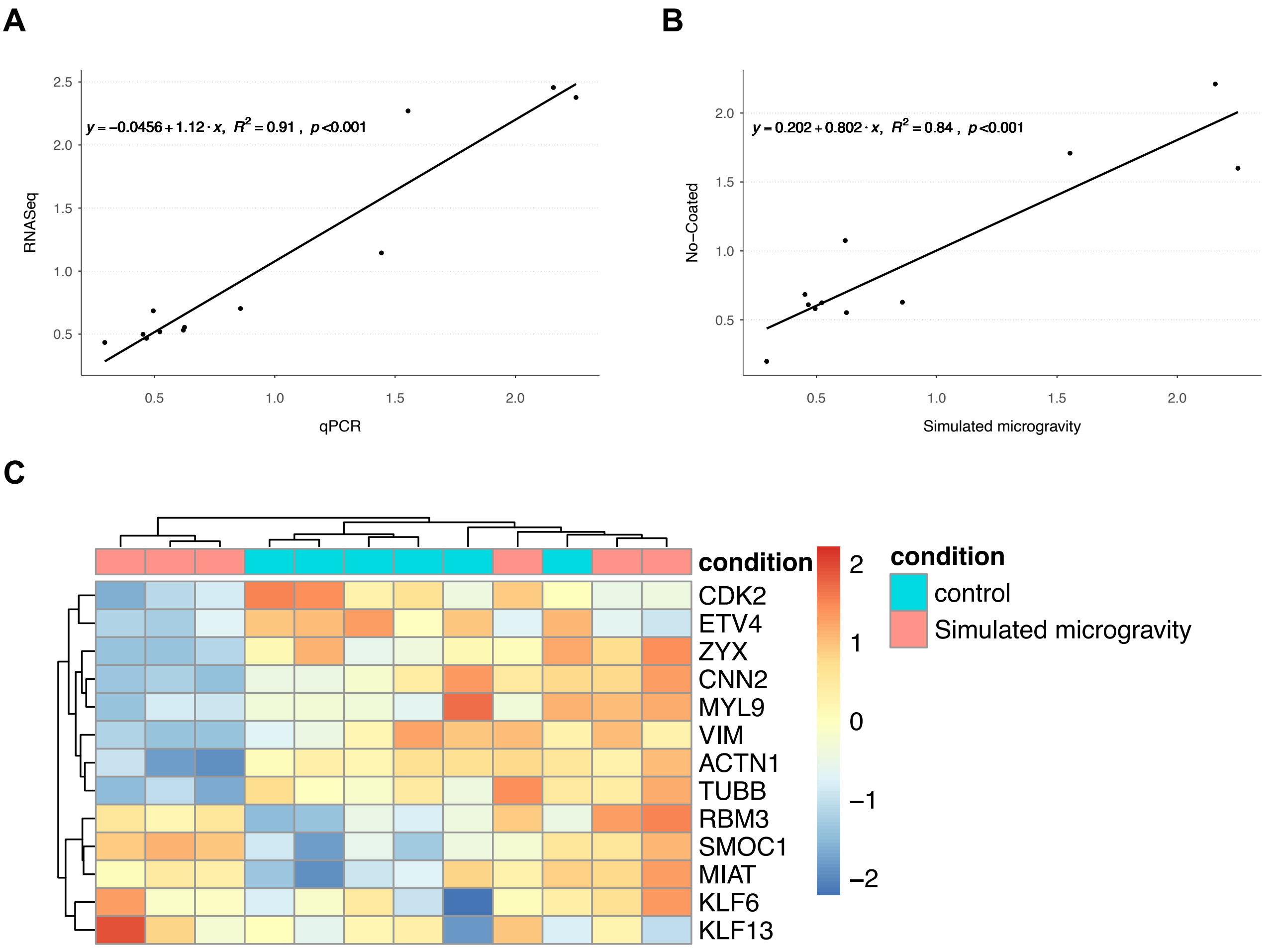

Figure S4, Correlation between RNA-Seq and qPCR results for the selected genes that came from the cells in the microgravity condition. **B,** Correlation of genes relative expression levels between in microgravity condition and in the no-coated (absence of laminin) condition. **C,** Heatmap showing the scaled expression levels (z-score) of RNA-Seq data of the selected genes evaluated by qPCR.

Figure S5

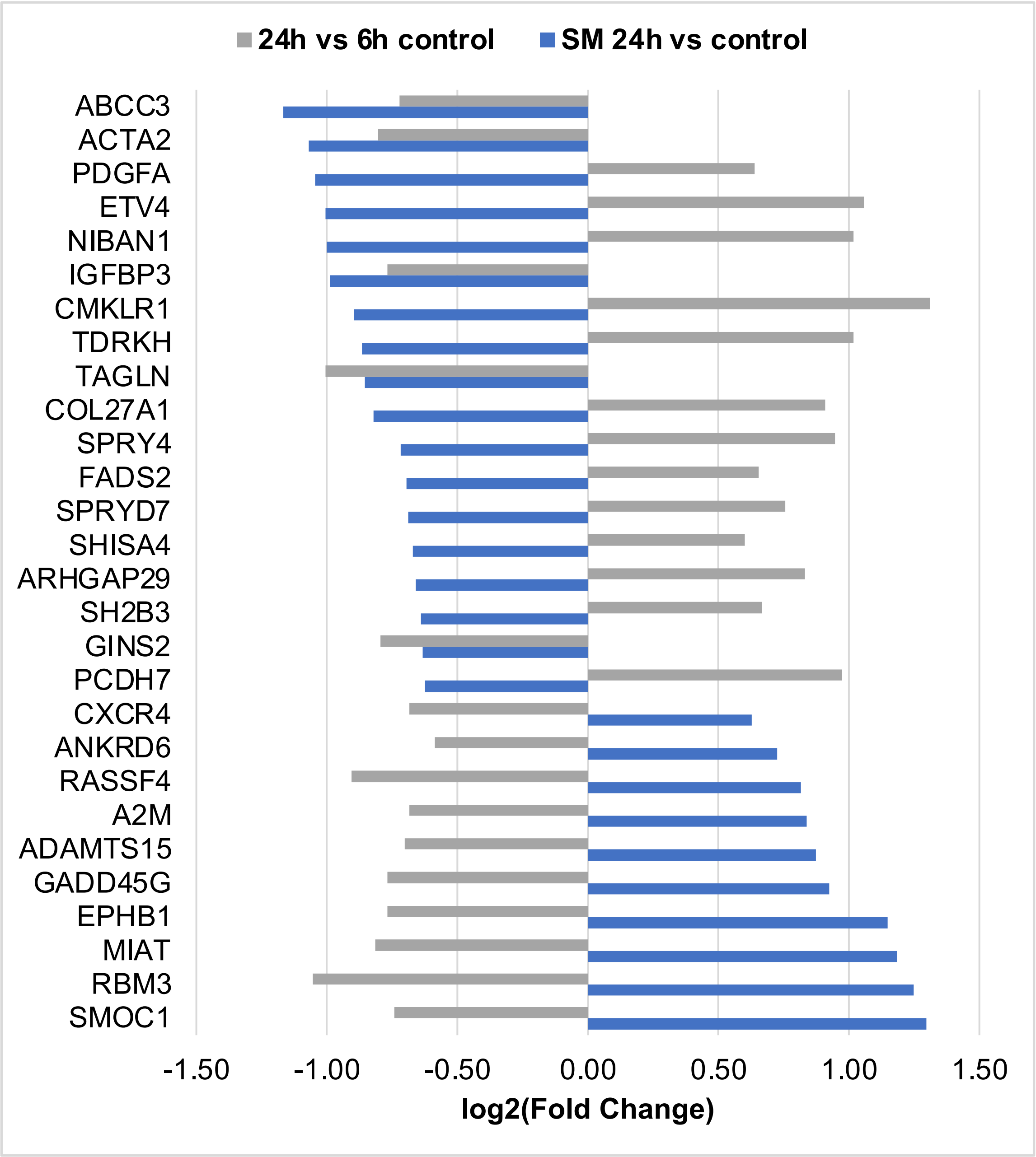

Figure S5, Comparison of log2(Fold Change) of significantly DEGs obtained from comparison of samples after 24h in simulated microgravity (SM) (vs sham control) and a comparison between controls kept at normal gravity in agitation for 24h samples vs 6h samples.

Figure S6

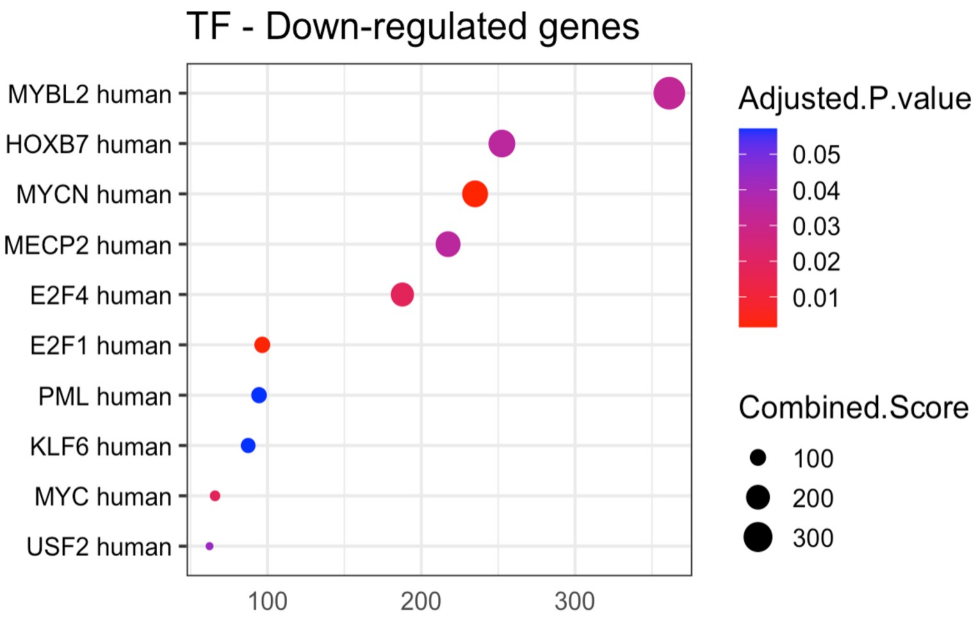

Figure S6. Prediction of Transcription factors potentially involved in regulation of downregulated DEGs in hNSPC exposed to 24h of simulated microgravity
